# Acquired Platelet Dysfunction with Eosinophilia: A Narrative Review

**DOI:** 10.3390/pediatric18030066

**Published:** 2026-05-07

**Authors:** Anselm Chi-Wai Lee

**Affiliations:** 1 Paediatric Haematology and Oncology Centre, Hong Kong Sanatorium and Hospital, Hong Kong; anselm.cw.lee@gmail.com; 2 Haematology and Oncology Centre, Department of Paediatrics and Adolescent Medicine, Hong Kong Children’s Hospital, Hong Kong

**Keywords:** acquired platelet dysfunction with eosinophilia, blood platelet disorders, eosinophilia, grey platelet syndrome, idiopathic purpura with grey platelets, thrombocytopenia

## Abstract

**Background**. Acquired platelet dysfunction with eosinophilia (APDE) is a transient bleeding disorder initially thought to occur exclusively in Southeast Asia. There are no uniformly agreed diagnostic criteria, and its full clinical features have not been defined. **Methods**. A literature search was conducted through MEDLINE, EMBASE, and Google Scholar for publications on APDE in order to explore patient demography, epidemiology, diagnostic criteria, and laboratory findings of the disease. **Results**. Ten retrospective, observational studies, five case series, and 21 case reports were identified with a total of 431 patients. Diagnostic criteria varied, with a two-tier approach for the diagnosis of impaired platelet function. In recent years, cases of APDE have been reported extensively outside of the Malay peninsula. Male patients (243/390, 62.3%) predominated. Their ages ranged from 11 months to 30 years, with only 40 (9.3%) subjects aged 18 years or older. Eosinophilia was absent in 10 to 33% of subjects in a few observational studies. Thrombocytopenia was present in 42 (9.7%) subjects. Parasitic infestation was less common in the new millennium. Spontaneous recovery within six months was the trend, and serious complications were extremely rare. **Conclusions**. APDE is no longer restricted to Southeast Asia. A uniform set of diagnostic criteria is needed. Clinician awareness followed by reliable but easily available laboratory tests is essential to confirm diagnosis. It is proposed that rapid diagnosis can be accomplished by screening the blood smear for grey platelets by a trained examiner followed by confirmatory platelet aggregometry. National or international collaboration and prospective studies are required to delineate the core and variable clinical and laboratory features of APDE.

## 1. Introduction

Acquired platelet dysfunction with eosinophilia (APDE) refers to a transient bleeding disorder, characterised by spontaneous, generalised mucocutaneous haemorrhage, that was first described in Southeast Asia some 50 years ago. According to the Singapore literature, the condition was first recognised in 1966 as a hitherto undescribed platelet disorder in children [1]. However, it was not until 1975 that Mitrakul first published the bleeding disorder based on the clinical and laboratory findings from 24 children from Chulalongkorn Hospital, Bangkok, Thailand [2]. In 1979, Suvatte et al. [3] from the Siriraj Hospital and Hathirat et al. [4] from the Ramathibodi Hospital published their experience with additional cases from Bangkok. They both coined the name APDE, which eventually outlasted other names suggested by Singaporean researchers in the following decade [1,5,6,7].

The early studies from Thailand showed that APDE occurred almost exclusively in children, lasted a few months, and eventually resolved invariably [2,3]. Bleeding manifestations were mild, and medical interventions were not necessary. Attempts to correct the platelet function abnormalities with corticosteroids, antihistamines, transfusion with fresh frozen plasma, and cryoprecipitate were not successful [2,4]. Platelet transfusion was the only effective treatment, if needed [4]. Symptomatic, life-threatening, or incessant bleeding that required platelet transfusion therapy was rare. No mortality was reported. When investigated, platelet count and coagulation screens were normal. Prolonged bleeding time in about 70% of patients suggested an underlying platelet function disorder [2,3]. This was supported by an impaired platelet release of adenosine diphosphate or triphosphate upon exposure to an agonist, and abnormal results on light transmission platelet aggregometry. More global tests on platelet function with clot retraction were often normal. Thus, a platelet storage pool disorder was thought to be the underlying haemostatic defect. Besides haemostatic studies, eosinophilia was almost always present [2,3]. Hence, APDE appeared to present itself as the mysterious bleeding disorder.

Prior to the end of the twentieth century, additional studies and case reports emerged [1,5,6,7,8,9,10,11,12,13], which raised more questions than answers. Cases of APDE were reported exclusively in Malaysia, Singapore, and Thailand, a narrow strip of three neighbouring countries that border the western South China Sea on the Malay Peninsula. While APDE affected children in Malaysia and Thailand, the disease only involved young adults and a couple of teenagers in Singapore [1,7,8,10]. In one Thailand hospital, APDE was so common that it outnumbered children with immune thrombocytopenia [3]. With more cases reported from other regions of the world in the 21st century, such peculiarities were no longer evident. Nevertheless, the unusual epidemiological observations suggest that each single centre may be biassed to certain aspects of APDE, and thus the true nature of the disease has never been defined. Therefore, the current literature review was undertaken to examine the known, the controversial, and the unexplored aspects of APDE.

## 2. Materials and Methods

A literature search was conducted across the databases of MEDLINE (from 1946) and EMBASE (from 1974) through to 18 December 2025 with the terms “acquired platelet dysfunction with eosinophilia” OR “idiopathic purpura with grey platelets” OR “eosinophilic purpura”. Additional searches were performed by going through the citations from selected publications and from Google Scholar for non-PubMed-indexed publications. The target was to be as broad as possible to gather all clinical cases with sufficient data. Commentaries and cases not diagnosed as APDE were excluded. Observational studies (with more than 10 cases), case series (with four to 10 cases), and case reports (with one to three cases) published in full, and observational studies published in conference abstracts, were included. However, when there was more than one publication from the same institution, only the latest work was selected for data analysis.

The following data were extracted from the selected publications for analysis: patient’s sex, age, clinical manifestations, physical findings, complete blood count, microscopic platelet morphology, bleeding time, laboratory tests for platelet function, investigations for parasite infestation, and the year and region of publication. The period of study was arbitrarily defined as the year 2000 and preceding years (twentieth century), and the period after the year 2000 (twenty-first century). Paediatric patients were aged under 18 years, and adult cases were 18 years and older. Eosinophilia was defined by an absolute eosinophil count > 0.5 × 10^9^/L. Anaemia was defined by haemoglobin < 11.0 g/dL, leukocytosis by total white blood cell (WBC) count > 15.0 × 10^9^/L, and thrombocytopenia by platelet count < 150 × 10^9^/L.

An attempt to search for a set of criteria for the diagnosis of acquired platelet dysfunction with eosinophilia as offered by the observational studies was made. The findings were compared with the objective analysis from the data extracted from the selected studies. From countries where APDE had been reported in both the twentieth and twenty-first centuries, changes in the epidemiology including sex, age, and prevalence of parasitic infestation were examined. Fisher’s exact test was employed when non-parametric variables were compared. No artificial intelligence-assisted technologies were used.

## 3. Results

**Figure 1:**
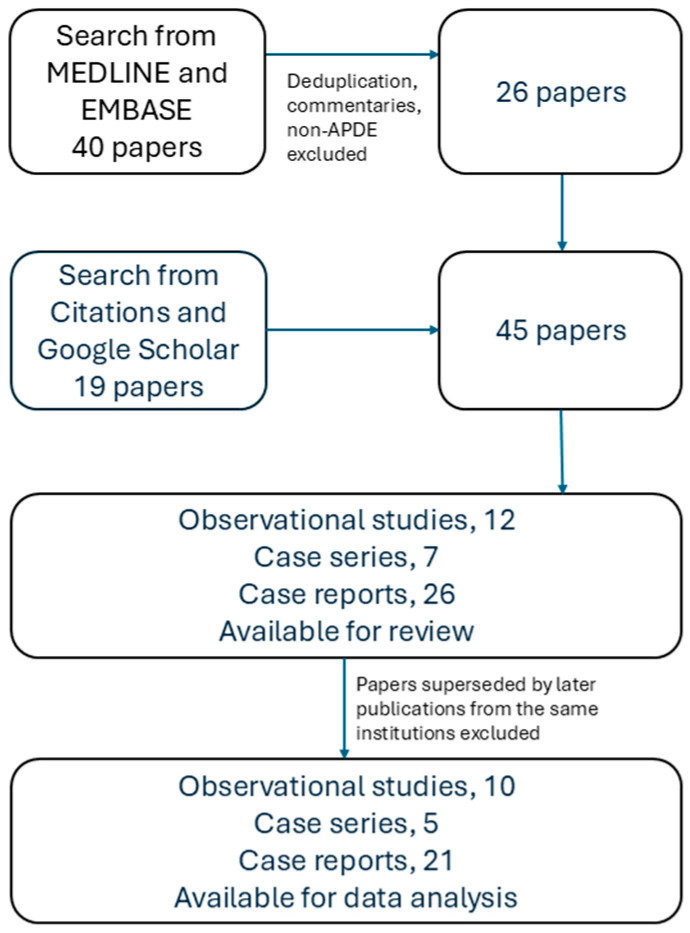
The search and selection process from the medical literature.

After deduplication and exclusion of 14 publications that did not match the inclusion criteria, the search from MEDLINE and EMBASE yielded 26 articles. The other searches found 19 articles. These 45 papers comprised 12 observational studies (eight full papers [1,2,3,11,14,15,16,17] and four conference abstracts [18,19,20,21]), seven case series [4,7,9,10,12,22,23], and 26 case reports [5,6,8,13,24,25,26,27,28,29,30,31,32,33,34,35,36,37,38,39,40,41,42,43,44,45]. Two observational studies [20], two case series [4,12] and five case reports [5,6,25,35,42] were not included for data analysis because they had been included in later studies from the same respective institutions. In particular, an observational study was excluded as the data presented did not match with a subsequent publication from the same institution. Laosombat et al. [14] reported 168 cases in 2001 without specific inclusion and exclusion criteria, and the study period was not mentioned. When Chotsampancharoen et al. [15] updated their research in 2018, taking into account patients diagnosed from 1981 to 2016 inclusively, their patient cohort grew to 307. However, 77 were excluded because of incomplete follow-up and laboratory data. Another 161 patients were excluded because their defining tests with bleeding time were found to be normal or absent. The more recent study ended up with only 69 patients. Therefore, the data presented in the earlier study by Laosombat et al. [14] were considered unreliable and excluded from further analysis. The comparison can be found in Table S1 in the Supplementary File. The publication search and selection processes are outlined in Figure 1.

The various criteria on which the diagnosis of APDE was based were extracted from the 10 observational studies and listed in Table 1. The only common criterion was a bleeding tendency characterised by spontaneous mucocutaneous haemorrhage. Seven studies listed eosinophilia with various thresholds. Prolonged cutaneous bleeding time, abnormal platelet morphology, and abnormal platelet function were mentioned separately in two studies each, respectively. Three studies also included a minimum platelet count as an inclusion criterion.

The total number of patients included in the selected 10 observational studies, five case series, and 21 case reports was 431. The clinical and laboratory features are summarised in Tables S2–S4 in the Supplementary File. Among those whose gender was known, there were 243 (62.3%) males and 147 (37.7%) females. Among the studies in which the exact ages were specified, the youngest was 11 months and the oldest was 30 years old. The great majority were paediatric patients, with 40 (9.3%) subjects aged 18 years or above. Before 2001, a total of 175 (40.6%) cases were reported across Canada [34], Hong Kong [39], Malaysia [9,13], Singapore [1,7,8,10], Thailand [2,3,11,15,36], the United Kingdom [28], and the United States [43]. Since 2001, 256 (59.4%) cases were reported across Albania [26], India [18,19,21,27,30,38,41], Malaysia [31,32,37], the Philippines [29], Singapore [22,40,44], Sri Lanka [16,17,24], Taiwan [33,45], Thailand [15], and Venezuela [23]. While the four children reported from the United Kingdom and North America were imported cases from Southeast Asia, the rest, with four exceptional cases [26,33,45], were considered residents in the tropical regions from where they developed APDE. The exceptional cases were reported in Albania and Taiwan in temperate zones. Although all the reporting regions were situated in the Northern Hemisphere, two cases reported by Lee were residents in Indonesia from the Southern Hemisphere [22].

With respect to clinical manifestations, mucocutaneous bleeding was consistently remarked to be present in all the publications. However, only the recent studies from India employed the International Society of Thrombosis and Haemostasis Bleeding Assessment Tool (ISTH-BAT) [46] as an objective instrument to define the bleeding diathesis [18,19].

Among the 30 patients described from 21 case reports, eosinophilia was universally present. Among the 37 cases reported from five case series, the absolute eosinophil count was normal in a single (2.7%) patient [22]. Among the 10 observational studies, 354 out of the 364 subjects had eosinophil counts recorded, which was normal in 14 (4.0%) patients [2,11,15]. However, it should be noted that patients with normal eosinophil counts were only reported from Thai studies in contrast to non-Thai studies (14/186 vs. 0/168, *p* = 0.0001; 95% CI for risk ratio, 0.8876–0.9634).

Besides eosinophilia, abnormalities in the rest of the complete blood count were uncommon. Two (5.5%) out of 36 patients mentioned in case reports and case series had anaemia [36,37] (Table S4 in Supplementary File). Both were children, with an 11-year-old male presenting with excessive bleeding after tooth extraction. Eleven (22.4%) out of 49 patients had leukocytosis (Table S4), often because of extreme eosinophilia [10,23,30,31,33,37,39,45]. Of note, 42 (9.7%) out of the 431 patients were reported to be thrombocytopenic [1,7,10,15,18,19,22,23,33,36,39,40,45] (Tables S2 and S4). Among the 19 cases with recorded platelet counts, 14 had mild thrombocytopenia (platelet 100–149 × 10^9^/L), four had moderate thrombocytopenia (50–99 × 10^9^/L), and one had severe thrombocytopenia (<50 × 10^9^/L).

The most popular tests for platelet dysfunction were cutaneous bleeding time and light transmittance platelet aggregometry (Tables S2 and S3). Other modalities such as platelet release of adenosine diphosphate or triphosphate, the Platelet Function Analyzer-100/200 instrument, and flow cytometry were sparingly used. The cutaneous bleeding time was recorded as prolonged in 303 (79.7%) of the 380 subjects tested. From the platelet aggregometry, abnormal aggregation was observed in 186/199 (93.5%) patients with adenosine diphosphate, 205/252 (81.3%) with collagen, 125/149 (83.9%) with epinephrine, 83/143 (58.0%) with arachidonic acid, and 14/158 (8.9%) with ristocetin.

Abnormal platelet morphology under the microscope in the form of grey or hypogranular platelets was consistently observed in five observational studies [11,15,18,19,21], found in 186 (95.4%) out of 195 subjects examined. Among the five case series, grey platelets were consistently observed in 10/10 (100%) children in one study [22], but was not specifically looked for in the other four publications. Eight patients from five case reports were also noted to have grey platelets [24,29,32,38,41]. One of these cases was a 6-year-old male reported by Mohamad et al. [32]. The peripheral blood smear was illustrated under magnification ×10 and ×40 and the platelets were remarked to be of normal granularity in the original report. However, when the photomicrograph was further magnified, 12 platelets were present and nine (75%) of them did not contain any granules.

Parasitic infestation was found in 98 (46.4%) out of 211 subjects tested (Tables S2 and S3). The parasites included Ancylostoma duodenale (n = 30), Ascaris lumbricoides (n = 21), Enterobius vermicularis (n = 17), Toxocara canis (n = 13), Trichuris trichiura (n = 5), Strongyloides stercoralis (n = 3), Filaria species (n = 2), and mixed infestation (n = 7). Other tests such as antinuclear antibody and serum immunoglobulin E were not routinely performed (Table S4).

The overall proportion of female patients with acquired platelet dysfunction with eosinophilia increased in the twenty-first century compared to the last millennium (103/256 vs. 44/134; *p* = 0.152), but the difference was not statistically significant. An exception was seen in Singapore where the male predominance was no longer evident in the twenty-first century (27/31 vs. 6/12, *p* = 0.017; 95% CI for odds ratio, 0.0316–0.6937).

In Singapore, adult cases predominated in the last century compared with the twenty-first century (29/31 vs. 0/12, *p* < 0.0001; 95% CI for risk ratio, 0.0169–0.2465). This is contrary to the rest of the world where adult cases were only seen in recent years (0/103 vs. 11/244, *p* = 0.038; 95% CI for risk ratio, 1.019–1.076).

The proportion of patients with APDE harbouring parasitic infestations remained the same before and after the year 2000 (58/115 vs. 40/96; *p* = 0.203). However, amidst the Southeast Asian countries (Malaysia, Singapore, and Thailand) where APDE was consistently reported in both periods, there has been a significant drop in the proportion of patients with parasitic infections in recent years (58/109 vs. 16/54, *p* = 0.004; 95% CI for odds ratio, 0.5013–0.8663).

With respect to clinical outcomes, spontaneous resolution has been uniformly reported, with duration of illness generally lasting from two to six months [1,3,16], with 10% of patients having symptoms for more than six months [15]. No intracranial haemorrhage or mortality were reported. However, there were no recommendations on how patients with APDE should be monitored after a diagnosis was made.

## 4. Discussion

Acquired platelet dysfunction with eosinophilia remains an obscure bleeding disorder known mainly by the paediatric discipline. It is unknown in standard textbooks [47,48]. Geographically, it is only restricted to a small part of the world. The condition was first reported in Thailand and, to date, 197 (45.7%) of the 431 cases of APDE identified from the medical literature were reported in this single country. However, case definition remains an unresolved issue, with different criteria employed by individual hospitals (Table 1), or even within the same institution at different times [14,15]. Hence, there is an immediate need for the centres where cases are frequently seen to agree on the core and variable clinical and laboratory components of APDE.

Acquired platelet dysfunction with eosinophilia implies that the disorder is non-hereditary in origin, characterised by a bleeding tendency because of thrombocytopathy and the presence of excessive eosinophils. This is similar to idiopathic thrombocytopenic purpura, a more common platelet disorder in childhood in which thrombocytopenia is mandatory for diagnosis. The non-hereditary origin is supported by the natural course of the illness with spontaneous resolution in all reported cases. The bleeding tendency is also supported by the universal observation of mucocutaneous bleeding from all the publications, although the means of identifying platelet dysfunction vary. Eosinophilia, however, appears to be a dispensable condition. When Hathirat et al. [11] reported 41 cases of APDE in 1993, four patients had normal eosinophil counts while 10 cases did not even have a recorded differential white cell count. Nonetheless, the paper was published with only 66% of the patients having eosinophilia. Laosombat et al. published the largest collection of patients in 2001 with 168 cases of APDE [14]. In that study, 23 (14%) patients did not have eosinophilia. The latest study was published by Chotsampancharoen et al. in 2018 [15]. Nine (13%) of the 69 cases had normal eosinophil counts, as deduced by the mean (3.0) and standard deviation (2.1) of the total sample stated in the paper. The discrepancy between the diagnostic label and the clinico-laboratory findings was first raised by Lee in 2017 [35], who demonstrated that the acquired bleeding disorder with platelet dysfunction could occur in children with no, mild, or severe eosinophilia, and that eosinophilia often persisted despite the resolution of bleeding and platelet abnormalities. Thus, eosinophilia is a common, but not mandatory, feature of APDE [49]. Nevertheless, severe eosinophilia (>1.5 × 10^9^/L) is often the first laboratory clue to the diagnosis of APDE (Table S3 in Supplementary File).

The haemostatic defect expressed by APDE is most probably due to a platelet storage pool disorder as shown from delicate laboratory procedures some 40 years ago [1]. However, no standard test has been recommended for its diagnosis, and no single test can reliably distinguish APDE from other bleeding diatheses. Based on the published literature, the tests for platelet dysfunction can be separated into two strata: an initial screening test, followed by a more specific evaluation of platelet function. Screening tests include the microscopic findings of grey or hypogranular platelets in >50% of the visible thrombocytes [15,22,41], a prolonged bleeding time, and abnormal closure time with adenosine diphosphate or epinephrine on the Platelet Function Analyzer-100/200 [18,19]. The Platelet Function Analyzer-100/200, however, is not a commonly available device in most service laboratories. The use of cutaneous bleeding time has also fallen out of favour in view of its invasive nature and poor reproducibility [50]. Microscopic examination of the peripheral blood smear, however, is a simple and almost universally available test. The finding of grey platelets is fairly sensitive (95.6%) and specific in the diagnosis of APDE as the haemato-morphology has only rarely been seen in other disorders such as hereditary grey platelet syndrome [51], or as a laboratory artefact associated with sample storage in ethylenediaminetetraacetic acid (EDTA) [52]. But, for the same reason that grey platelets are rarely encountered, laboratory staff who are responsible for examination of the blood smear have to be properly trained to look for the abnormality under oil emersion at ×100 magnification. Otherwise, the condition can be easily missed [32]. That said, the number of cases with grey platelets that correlated directly with impaired platelet aggregation is still limited. Further prospective studies are needed to support this approach.

Confirmatory tests for platelet storage pool disorder are commonly accomplished by light transmission platelet aggregometry as found in this literature review. Abnormal platelet aggregation following exposure to adenosine diphosphate, collagen, and epinephrine, alone or in combination, is practically seen in all cases. Coincidentally, the same pattern of abnormalities is also seen in grey platelet syndrome [51]. Other investigations such as electron microscopy [14] and tests of platelet release of adenosine diphosphate or triphosphate for adhesion responses are rarely conducted [2,3,21]. Hence, screening for grey or hypogranular platelets at the time when the first complete blood count is obtained, followed by light transmission platelet aggregometry as a confirmation test, is the most feasible approach to establish a diagnosis of APDE in most clinical settings. Further observations and studies will inform if the microscopic features of grey platelets are a core or variable component of the clinical entity.

The underlying pathogenesis of APDE remains unexplained. In the early period, a common theme emerged and suggested APDE as part of an allergic reaction to intestinal helminths [1,3,7,14]. Some even called it allergic vascular purpura or allergic purpura [3,11]. This was widely accepted in view of the prevalence of eosinophilia and helminthic infestations at those times. However, with the current understanding that at least 10% of patients with APDE are not eosinophilic and the uncommon occurrence of parasitic infestations in recent times, the allergic basis as a causation of APDE remains unproven. The recent expansion of the clinical spectrum in the hereditary grey platelet syndrome to include immune dysregulation and autoimmunity could suggest the loss of platelet α granules as the primary event in APDE [53].

The current review also suggests that APDE and immune thrombocytopenia (ITP, previously idiopathic thrombocytopenic purpura) may not be completely separate entities. Of note, almost 10% of patients in the literature had thrombocytopenia. Although the majority of thrombocytopenia are categorised as mild, the skewing of platelet counts towards the mild end may be a biassed observation. First, with moderate or severe thrombocytopenia, ITP will more likely be diagnosed rather than APDE as the former is more common and better known. Second, conventional tests for platelet function are less sensitive in the presence of thrombocytopenia and are generally not recommended when the platelet count is below 100 × 10^9^/L [54]. On the other hand, among the limited studies of platelet function in ITP, Stafylidis et al. observed impaired platelet aggregation in response to adenosine diphosphate and collagen while the reactivity to ristocetin was preserved [55]. This pattern of abnormality is comparable to that of APDE. Hence, further studies to characterise the relationship between APDE and ITP are warranted but, at present, they should not be viewed as mutually exclusive disorders (Table 2). Indeed, Poon et al. were able to demonstrate the presence of platelet-associated immunoglobulin G in one of the two patients they reported, and suggested that the bleeding disorder and eosinophilia were independent events secondary to a common immunological phenomenon [34].

The clustering of APDE cases in a group of young men in Singapore during the last century was intriguing. They were almost always national servicepeople and exclusively male recruits [1,7,8,10]. The disease seems to have disappeared in adults from Singapore recently. Clustering within a family is extremely rare [13], and there have not been any reports of clustering in child care facilities or schools despite APDE being a predominantly childhood illness.

Patients diagnosed with APDE are often offered general precautions similar to ITP. No specific treatments for the improvement of platelet function have been found. Indeed, more than 90% of patients recover spontaneously without treatment within six months. The majority of studies reported the use of anti-helminthic therapy irrespective of the finding of parasites, but equally successful outcomes have been noted in the absence of deworming treatment [22]. With the changing epidemiology and fewer patients found to have helminthic infestations, empirical treatment may not be justified.

There are no systematic studies to inform how patients diagnosed with APDE should be monitored during follow-up. In the author’s practice, patients are followed up at monthly intervals, physically or online, until disease resolution. Repeat blood tests are performed when clinical condition changes or upon discharge. Clinical assessment using a standardised scoring system such as the Buchanan scores [56] or the ISTH-BAT scores [46] has not been used or tested prospectively. Eosinophilia does not appear to be relevant as a significant proportion of patients remain eosinophilic when their bleeding manifestations have subsided [14,15,22]. The enumeration of grey platelets on the peripheral blood smear can be a simple means of estimating disease activity [22,35], while platelet aggregometry is a more sophisticated way of measuring disease resolution and remission.

As a last note, the name APDE should be re-evaluated. Taking into account all the case series and case reports, and some of the recent observational studies, it can be seen how the name APDE has resulted in a feedback loop failure in portraying the true clinical spectrum of the disease [57]. First, with just one exception, all the patients had eosinophilia as predicted by the name. Second, patients with thrombocytopenia are not excluded because the name APDE does not restrict this feature. Third, in the study by Chin and Koong [9], eight patients were found to have platelet storage pool disorder but only seven were diagnosed with APDE. The remaining patient could have been wrongly excluded just because eosinophilia was absent. Therefore, it is possible that the name APDE has resulted in an incorrect conceptual framework and determined how future cases would look like, degrading the true clinical spectrum of the bleeding disorder [57]. In this respect, idiopathic purpura with grey platelets has been suggested as an alternative [22,35], but the name can only be valid if grey platelets are a universally true laboratory feature in prospective studies.

## 5. Limitations

The study is limited by the low quality of the published studies. The best available evidence comes only from retrospective, single-institution studies. Comparisons and pooling of data are questionable as case definitions are highly variable across the studies. Potential bias is possible given that the majority of the included publications are case reports. Hence, the pooled data as presented in this study should be treated with caution. As an explorative study for APDE and a call for prospective, collaborative studies, a narrative review suffices. Further steps into the quality assessment of the included studies can differentiate the weight of evidence from observational studies versus case reports. Thus, scoping or systematic reviews may be more informative but resource limitations do not permit in-depth analysis.

## 6. Conclusions

APDE is a transient bleeding disorder characterised by platelet storage pool defects. It should be suspected when a previously well patient presents with abnormal mucocutaneous bleeding, not commensurate with the prevailing platelet count. A quick screening can be accomplished by examination of the peripheral blood smear for grey platelets by trained personnel. A second-tier test with light transmission platelet aggregometry can follow for confirmation. The presence of eosinophilia, especially moderate to severe eosinophilia, can be a useful clue. The presence of thrombocytopenia, especially mild thrombocytopenia, does not exclude the diagnosis. Further studies and regional collaborations are needed to investigate the underlying pathogenesis and unify diagnostic and management strategies. Epidemiology has changed and the disease is no longer restricted to Southeast Asia or the tropical regions.

## Figures and Tables

**Table 1:** Diagnostic criteria for acquired platelet dysfunction with eosinophilia as stated by the observational studies.

**Studies**	**Diagnostic Criteria**	**Contradictions**
Mitrakul [2]	(a) Bleeding tendency	Nil
Suvatte [3]	(a) Bleeding tendency	Nil
Kueh [1]	(a) Bleeding tendency (b) Eosinophilia	Nil
Hathirat [4,11]	(a) Bleeding tendency (b) Platelet > 100 × 10^9^/L (c) Eosinophilia (d) Prolonged bleeding time (e) Abnormal platelet morphology and function	In their latest publication with 41 patients, eight had normal eosinophil counts and 10 did not have documented eosinophil counts.
Wickrammasinghe [17]	(a) Bleeding tendency (b) Eosinophil > 1 × 10^9^/L	Nil
Lucas [16]	(a) Bleeding tendency (b) Eosinophilia (c) Normal platelet count	Nil
Sukumaran [21]	(a) Bleeding tendency (b) Eosinophilia (c) Normal platelet count	Nil
Chotsampancharoen [15]	(a) Bleeding tendency (b) Eosinophil > 0.5 × 10^9^/L (c) Abnormal platelet morphology (d) Prolonged bleeding time	Nine out of the 69 subjects had eosinophil < 0.5 × 10^9^/L. In a prior report from the same hospital, 14% of subjects had normal eosinophil counts.
Dave [18]	(a) Bleeding tendency	Nil
Thangaraja [19]	(a) Bleeding tendency (b) Eosinophil > 0.5 × 10^9^/L (c) Abnormal platelet function	Nil

**Table 2:** Overlapping and differential features between acquired platelet dysfunction (APDE) and acute/persistent immune thrombocytopenia (ITP).

	ITP, Acute or Persistent	APDE
Age	All ages	Childhood illness
Male:Female	Approximately 1.2:1	Approximately 1.5:1
Manifestations	Mucocutaneous bleeding; life-threatening bleeding < 1%	Mucocutaneous bleeding; life-threatening bleeding not reported
Outcome	Universal recovery	Universal recovery
Eosinophilia	Commonly absent	Commonly found
Thrombocytopenia	100%	Approximately 10%
Anti-platelet antibodies	Common but not universal	Only tested and found in a single case
Platelet dysfunction	Found in some patients; systematic studies are lacking	Consistent with storage pool disorder
Response to steroid	Yes	No

## Data Availability

No new data were created or analyzed in this study.

## Supplementary Materials

The following supporting information can be downloaded at: https://www.mdpi.com/article/10.3390/pediatric18030066/s1. Table S1: Comparison of the studies published by Laosombat et al. and Chotsampancharoen et al.; Table S2: Clinical and laboratory summary of the observational studies on acquired platelet dysfunction with eosinophilia. Table S3. Clinical and main laboratory features of the case series and case reports of acquired platelet dysfunction with eosinophilia. Table S4. Other laboratory features of the case series and case reports of acquired platelet dysfunction with eosinophilia.

## Abbreviations

The following abbreviations are used in this manuscript:

APDE	Acquired platelet dysfunction with eosinophilia
EDTA	Ethylenediaminetetraacetic acid
ITP	Immune thrombocytopenia (or immune thrombocytopenic purpura)
