# Supplementary material for: Acquired Platelet Dysfunction with Eosinophilia: A Narrative Review
Source: Pediatr Rep. 2026 May 7;18(3):66. doi: 10.3390/pediatric18030066 (PMC13214754; doi:10.3390/pediatric18030066)
Supplement: Supplementary file 1 [file pediatrrep-18-00066-s001.zip › pediatrrep-4273109-supplementary.pdf]

Supplementary file

Table S1. Comparison of the studies published by Laosombat et al. and Chotsampancharoen et al.

	Laosombat et al.	Chotsampancharoen et al.
Year of publication	2001	2018
Institution	Prince of Songkla University, Thailand	Prince of Songkla University, Thailand
Study design & Patient selection		
Inclusion criteria	Children with widespread spontaneous bruising on the extremities, body and face	Children with widespread spontaneous bruising on the extremities, body and face
Study period	Not mentioned	1981 – 2016, inclusively
No. of patients included	168	307
Exclusion criteria	Prior history of bleeding tendency	Incomplete laboratory data (n=77)<br>Normal bleeding time, < 8 minutes (n=111)<br>Unmeasured bleeding time (n=50)
No. of patients excluded	Not mentioned	238
Final number of patients	168	69

Table S2. Clinical and laboratory summary of the observational studies on acquired platelet dysfunction with eosinophilia.

References	No. of patients	Sex/Age	Eosinophilia (>0.5 × 10^9/L)	Grey platelets	Prolonged Bleeding time	Platelet aggregation (abnormal/total tested)	Other platelet parameters (abnormal/total tested)	Worms/parasites
Mitrakul, 1975 (Thailand) [2]	24	M:F= 15:9/ 1.2 – 13.0 (mean 6.8) years	23/24 (95.8%)	ND	16/22 (72.7%)	COLL 6/15 (40.0%) Thrombin 4/14 (28.6%)	Clot retraction, 4/15 (26.7%) Platelet adhesiveness, 9/16 (56.3%) Platelet factor 3 release 2/15 (13.3%) ADP release, 12/14 (85.7%) Thrombocytopenia, 0	Positive results for Ascaris and Enterobius in only few cases
Suvatte, 1979 (Thailand) [3]	62	M:F= 35:27/ 1.6 – 11.0 (mean 6.5) years	100%	ND	42/62 (67.7%)	ADP 42/62 (67.7%) COLL 54/62 (87.1%) RISTO 6/8 (75.0%) Thrombin 43/54 (79.6%)	Clot retraction 0% Platelet adhesiveness 38/60 (63.3%) Platelet factor 3 release 25/50 (50.0%) Thrombocytopenia, 0	Ascaris 17 (27.4%) Enterobius 14 (22.6%) Ancylostoma 2 (3.2%)

Kueh, 1982, 1983, 1986 (Singapore) [1,5,6]	16	M:F= 13:3/ 14.0 – 22.0 (mean 19.3) years; (≥18= 14)	100%	ND	ND	ADP 1/16 (6.3%) COLL 11/16 (68/8%) EPI 15/16 (93.7%) ARACG 5/16 (31.3%)	Thrombocytopenia, 1 (6.3%)	Ancylostoma 7 (43.8%) Strongyloides 1 (6.3%)
Hathirat, 1979, 1982, 1993 (Thailand) [4,11,12]	41	NA	27/31 (87.1%)	Most	100%	ND	ND	Ancylostoma 3/13 Enterobius 3/13 Ascaris 2/13 Trichuris 1/13
Wickrammasinghe, 2001 (Sri Lanka) [17]	14	M:F= 11:3/ Paediatric	100%	ND	100%	ADP 3/5 (60%) COLL 4/5 (80%) ARACH 3/5 (60%) RISTO 2/5 (40%)	Thrombocytopenia, 0	Toxocara 12/14 (85.7%) Ascaris 2/14 (14.3%)
Lucas, 1996, 2002 (Sri Lanka) [16,25]	12	M:F= 7:5/ 1.3 – 10.0 (mean 4.8) years	100%	ND	7/12 (58.3%)	ADP 12/12 (100%) COLL 12/12 (100%) RISTO 3/12 (25.0%)	Thrombocytopenia, 0	Filarial serology positive in 2
Sukumaran, 2009 (India) [21]	38	M:F= 26:12/	100%	29/38 (76.4%)	25/38 (65.8%)	ADP 38/38 (100%)	Absent ATP release, 46.6%	ND

		0.9 – 10.0 years				COLL 38/38 (100%)<br>EPI 33/38 (86.9%)<br>ARACH 38/38 (100%)<br>RISTO 0%	Thrombocytopenia, 0%	
Chotsampancharoen, 2018 (Thailand) [15]	69	M:F= 44:25/<br>0 – 16 (mean 6.9) years	60/69 (87%)	100%	100%	ND	Thrombocytopenia, 10 (14.5%)	Ancylostoma 11/42 (26.2%)<br>Trichuris 3/42 (7.1%)<br>Multiple 2/42 (4.8%)
Dave, 2021 (India) [18,20]	54	M:F= 30:24/<br>5.0 – 12.0 (median 8.0) years	100%	100%	40/54 (74.1%)	ADP 49/54 (90.7%)<br>COLL 40/54 (74.1%)<br>EPI 43/54 (79.6%)<br>ARACG 22/54 (40.7%)<br>RISTO 0/54	Prolonged closure time on PFA200, 87%<br>Reduced ATP release, 62.5%<br>Thrombocytopenia, 9 (16.7%)	ND
Thangaraja, 2025 (India) [19]	34	M:F= 14:20/	100%	100%	23/34 (67.6%)	Abnormal in 91% of tested	Prolonged PFA-200, 62%<br>Thrombocytopenia, 3 (8.8%)	ND

		Median age 12.0 years (≥18= 9)					Bernard-Soulier syndrome, 2	
Total	364	M:F = 195:128	340/354 (96.0%)	186/195 (95.4%)	277/346 (80.1%)	ADP 145/187 (77.5%)<br>COLL 165/202 (81.7%)<br>EPI 91/108 (84.3%)<br>ARACH 68/113 (60.2%)<br>RISTO 11/117 (9.4%)	Thrombocytopenia, 23/364 (6.3%)	82/159 (51.2%)

Abbreviations/notes: Fractions = number of abnormal tests/total number of patients tested; ADP, adenosine diphosphate; ARACH, arachidonic acid; COLL, collagen; EPI, epinephrine; F, female; M, males; PFA, platelet function analyzer; RISTO, ristocetin.

Table S3. Clinical and main laboratory features of the case series and case reports of acquired platelet dysfunction with eosinophilia.

References	Country	Sex/Age	Eos	Gray Platelet	Bleeding time	Platelet aggregation					Helminths/ parasites	Remarks
						A	C	E	Aa	R		
Bayever, 1984 [43]	USA	M/12	4.86	-	Prolonged	↓	↓	↓	N	N	Negative	Travelled to Philippines
Muthiah, 1984 [28]	UK	F/8	1.66	-	Prolonged	N	↓	N	-	N	Negative	Imported from Malaysia
Teo, 1984 [7]	Singapore	F/21	4.6	-	Prolonged	N	↓	-	-	-	Ancylostoma	
		M/19	3.3	-	Prolonged	↓	↓	-	-	-	Negative	
		M/19	3.1	-	Prolonged	N	↓	-	-	-	Negative	
		M/20	3.3	-	Normal	↓	↓	N	-	-	Ancylostoma	
		M/19	2.1	-	Prolonged	-	-	-	-	-	Negative	
		M/19	2.0	-	Prolonged	↓	↓	↓	-	-	Negative	
		M/19	1.7	-	Prolonged	↓	↓	↓	-	-	Ancylostoma	
Ramanathan, 1987 [13]	Malaysia	F/8	1.73	-	Prolonged	-	-	-	-	-	Negative	
		M/10	0.85	-	Prolonged	↓	N	-	-	N	Ancylostoma	
		M/11	1.06	-	Prolonged	↓	N	-	-	N	Ancylostoma	
Lim, 1989 [10]	Singapore	M/19-22	4.6	-	-	↓	↓	↓	N	N	Negative	
		M/19-22	7.0	-	-	N	↓	N	↓	N	Negative	

		M/19-22	2.4	-	-	N	↓	N	↓	N	Negative	
		M/19-22	3.9	-	-	-	-	-	-	-	Strongyloides	
		M/19-22	1.9	-	-	↓	↓	↓	↓	N	Negative	
		M/19-22	4.0	-	-	N	N	↓	↓	N	Strongyloides	
		M/19-22	1.8	-	-	N	↓	↓	↓	N	Negative	
Chin, 1990 [9]	Malaysia	F/9	1.65	-	Normal	N	N	-	-	N	-	
		M/10	1.11	-	Prolonged	↓	N	-	-	N	-	
		M/5	2.66	-	Prolonged	↓	N	-	-	N	-	
		M/14	5.00	-	Prolonged	↓	↓	-	-	N	-	
		M/7	1.73	-	Normal	↓	N	-	-	N	-	
		M/9	1.88	-	Prolonged	↓	↓	-	-	↓	-	
		M/6	1.27	-	Normal	-	-	-	-	-	-	
Chinprateeb, 1992 [36]	Thailand	M/11	1.85	-	Prolonged	-	-	-	-	-	-	
Poon, 1995 [34]	Canada	M/5	1.5	-	Prolonged	-	-	-	-	-	Negative	Imported from Malaysia
		M/6	2.8	-	Prolonged	↓	↓	↓	↓	N	Negative	
Lim, 1999 [8]	Singapore	M/20	1.51	-	Prolonged	↓	↓	↓	↓	N	Ancylostoma	
Zhou, 2000 [39]	Hong Kong	M/4	3.5	-	-	N	N	↓	N	N	Negative	In Thailand 5 months ago
		F/8	8.86	-	-	N	↓	↓	N	N	Negative	In Nepal 6 months ago

Ruiz-Saez, 2005	Venezuela	F/11	2.37	-	Normal	↓	↓	↓	-	N	As+ Bl	
[23]		M/12	1.48	-	Prolonged	↓	↓	↓	-	N	Ne+Tr	
		M/12	1.62	-	Prolonged	↓	↓	N	-	N	St+Gi+Bl	
		F/3	1.66	-	Normal	↓	↓	↓	-	N	Trichuris	
		F/5	3.83	-	Prolonged	↓	↓	↓	-	N	Ne+Bl+As+En	
		F/9	8.90	-	Normal	N	↓	N	-	N	Ne+As+Tr	
Lee, 2012, 2017,	Singapore	M/11	7.40	Present	-	N	↓	↓	N	N	Negative	Resident in Indonesia
2019		M/8	3.17	Present	-	↓	↓	↓	↓	N	Negative	-
[22,35,42]		M/5	0.75	Present	-	↓	↓	↓	N	N	Negative	-
		F/3	0.29	Present	-	↓	↓	↓	N	N	-	-
		F/4	4.20	Present	-	↓	↓	↓	N	N	Negative	-
		M/5	2.82	Present	-	↓	↓	↓	N	↓	Negative	-
		M/4	2.03	Present	-	↓	↓	↓	N	N	Negative	-
		F/16	1.49	Present	-	-	-	-	-	-	-	-
		F/10	2.68	Present	-	↓	↓	↓	N	N	Negative	-
		M/13	5.01	Present	-	↓	↓	↓	N	N	Negative	Resident in Indonesia
Singh, 2014 [38]	India	M/11mo	Present	Present	-	↓	-	↓	↓	N	Negative	
		M/30	Present	Present	-	↓	↓	↓	↓	N	-	
		M/4	Present	Present	-	↓	↓	N	↓	N	-	
Chen, 2015 [45]	Taiwan	M/15mo	13.58	-	-	-	-	-	-	-	Ancylostoma	
Yadav, 2016 [30]	India	M/11	7.70	-	Prolonged	↓	↓	-	-	N	-	

Khan, 2017 [27]	India	M/8	3.2	-	Prolonged	↓	↓	↓	↓	-	Negative
Tan, 2018 [40]	Singapore	F/4	4.12	-	-	↓	↓	↓	↓	N	Negative
Shih, 2020 [33]	Taiwan	F/5	6.76	-	-	↓*	-	↓*	-	-	Negative
		F/6	5.62	-	-	↓*	-	↓*	-	-	Negative
Tang, 2020 [44]	Singapore	F/6	2.37	-	-	↓	N	↓	N	N	Negative
Villanueva, 2020 [29]	Philippines	M/6	3.05	Present	Normal	↓*	-	↓*	-	-	Negative
Anuar, 2022 [31]	Malaysia	M/3	2.61	-	-	-	-	-	-	-	Negative
Dharmabandu, 2023 [24]	Sri Lanka	M/14	2.28	Present	Prolonged	-	-	-	-	-	Toxocara
Koti, 2023 [26]	Albania	F/7	2.57	-	Prolonged	-	-	-	-	-	Negative
Saidin, 2024 [37]	Malaysia	F/9	9.90	-	-	-	↓	-	N	N	Negative
		M/23	1.17	-	-	-	↓	-	↓	↓	-

Mohamad, 2024 [32]	Malaysia	M/6	2.62	Present	-	-	-	-	-	-	-
Dahiya, 2025 [41]	India	M/4	2.24	Present	-	↓	↓	↓	↓	N	Negative
		M/10	1.98	Present	-	↓	N	↓	N	N	Negative
Total	67 patients	M:F = 48:19	-	18/18 (100%)	26/34 (76.5%)	41/52	40/50	34/41	15/30	3/44	16/52 (30.8%)

Abbreviations/Notes: Fractions = number of abnormal tests/total number of patients tested; -, Not done or mentioned; ↓, impaired aggregation; A, adenosine diphosphate; Aa, arachidonic acid; As, Ascaris lumbricoides; Bl, Blastocystis hominis; C, collagen; E, epinephrine; En, Enterobius vermicularis; Eos, eosinophil; F, female; Gi, Giardia intestinalis; M, male; mo, months old; N, normal; Ne, Necator americanus; St, Strongyloides stercoralis; Tr, Trichuris trichiura; R, ristocetin; UK, United Kingdom; USA, United States of America.

Table S4. Other laboratory features of the case series and case reports of acquired platelet dysfunction with eosinophilia.

References	Sex/Age	Hb	WBC	Neutrophil	Lymphocyte	Monocyte	Platelet	ANA	IgE
Bayever, 1984 [43]	M/12	13.9	11.3	3.28	2.37	0.45	233	Negative	*1510 U/mL*
Muthiah, 1984 [28]	F/8	12.1	7.2	2.66	2.80	0.08	212	Negative	-
Teo, 1984 [7]	F/21	-	-	-	-	-	290	-	-
	M/19	-	-	-	-	-	165	-	-
	M/19	-	-	-	-	-	*130*	-	-
	M/20	-	-	-	-	-	200	-	-
	M/19	-	-	-	-	-	*100*	-	-
	M/19	-	-	-	-	-	*105*	-	-
	M/19	-	-	-	-	-	*120*	-	-
Ramanathan, 1987 [13]	F/8	11.6	9.1	-	-	-	399	-	-
	M/10	13.0	7.7	-	-	-	194	-	-
	M/11	11.4	5.6	-	-	-	157	-	-
Lim, 1989 [10]	M/19-22	-	13.6	-	-	-	*85*	-	*416 IU/L*
	M/19-22	-	15.5	-	-	-	150	-	*195 IU/L*
	M/19-22	-	9.4	-	-	-	*70*	-	*>2000 IU/L*
	M/19-22	-	19.0	-	-	-	150	-	*>2000 IU/L*

	M/19-22	-	8.7	-	-	-	*35*	-	*654 IU/L*
	M/19-22	-	12.4	-	-	-	*140*	-	-
	M/19-22	-	8.2	-	-	-	*145*	-	-
Chinprateeb, 1992 [36]	M/11	*9.2*	10.9	-	-	-	*135*	-	-
Poon, 1995 [34]	M/5	11.0	7.4	2.74	3.11	0	284	-	
	M/6	12.6	9.5	2.57	3.14	0.48	274	-	
Lim, 1999 [8]	M/20	13.9	8.4	4.79	1.26	0.67	169	-	*2213 IU/L*
Zhou, 2000 [39]	M/4	12.5	12.7	-	-	-	*114*	Negative	-
	F/8	12.0	22.25	-	-	-	*129*	Negative	↑
Ruiz-Saez, 2005 [23]	F/11	-	10.3	-	-	-	184	-	-
	M/12	-	8.7	-	-	-	299	-	-
	M/12	-	8.5	-	-	-	*86*	-	-
	F/3	-	5.4	-	-	-	247	-	-
	F/5	-	21.3	-	-	-	351	-	-
	F/9	-	21.2	-	-	-	188	-	-
Lee, 2012, 2019 [22,42]	M/11	12.8	14.8	3.12	3.41	0.89	223	-	*>2000 IU/mL*
	M/8	13.1	12.69	5.58	2.79	0.76	219	-	-
	M/5	13.0	6.8	3.81	1.90	0.27	153	Negative	-

	F/3	11.1	9.74	2.92	6.23	0.29	173	-	-
	F/4	12.2	11.06	1.99	4.31	0.44	186	-	-
	M/5	11.2	10.46	4.08	3.24	0.31	210	-	-
	M/4	11.4	9.36	-	-	-	*146*	-	-
	F/16	12.0	9.17	3.35	3.57	0.69	*114*	-	-
	F/10	12.5	10.15	4.51	2.42	0.48	190	-	-
	M/13	14.6	13.59	4.12	4.02	0.29	*148*	-	-
Singh, 2014 [38]	M/11mo	-	-	-	-	-	Normal	-	-
	M/30	-	-	-	-	-	Normal	-	-
	M/4	-	-	-	-	-	Normal	-	-
Chen, 2015 [45]	M/15mo	11.4	28.0	2.94	10.42	0.98	*75*	-	*263 kU/L*
Yadav, 2016 [30]	M/11	13.8	22.0	7.70	5.50	0.88	264	-	-
Khan, 2017 [27]	M/8	-	-	-	-	-	-	-	Normal
Tan, 2018 [40]	F/4	12.1	14.72	5.59	4.27	0.44	*135*	-	-
Shih, 2020 [33]	F/5	12.6	16.1	6.76	1.77	0.81	210	Negative	*598 kU/L*
	F/6	12.3	15.14	4.68	4.44	0.41	*148*	Negative	*159 kU/L*
Tang, 2020 [44]	F/6	14.2	13.15	2.37	7.89	-	203	-	-

Villanueva, 2020 [29]	M/6	12.0	8.46	3.47	1.40	0.38	179	-	-
Anuar, 2022 [31]	M/3	13.1	16.0	-	-	-	200	-	-
Dharmabandu, 2023 [24]	M/14	12.5	9.62	-	-	-	233	-	-
Koti, 2023 [26]	F/7	11.9	9.4	2.72	3.42	0.61	327	-	-
Saidin, 2024 [37]	F/9	*9.0*	20.2	-	-	-	535	-	-
	M/23	12.9	10.15	-	-	-	194	-	-
Mohamad, 2024 [32]	M/6	12.5	10.84	-	-	-	203	-	-
Dahiya, 2025 [41]	M/4	11.6	6.23	-	-	-	216	-	-
	M/10	11.0	5.51	-	-	-	190	-	-
Total	M:F= 48:19 Mean age= 11.6	Hb<11 2/36 (5.5%)	WBC>15 11/49 (22.4%)				Plt<150 19/67 (28.4%)	Positive, 0/7	Elevated, 11/12 (91.7%)

Abbreviations: Fractions = number of abnormal tests/total number of patients tested; All differential counts in × 10^9/L; -, not done/mentioned; F, female; Hb, haemoglobin (g/dL); M, male; mo, months old; WBC, white blood cell (× 10^9/L).
